# Exercise and Cardiovascular Risk Modulation in Post-COVID Syndrome

**DOI:** 10.1007/s11883-026-01450-y

**Published:** 2026-08-13

**Authors:** Cássia da Luz Goulart, Leandro Tolfo Franzoni, Filipe Ferrari, Ananda Silveira Cardoso, Natália Bittencourt, Ricardo Stein

**Affiliations:** 1 Exercise Cardiology Research Group (CardioEx), Hospital de Clínicas de Porto Alegre, Universidade Federal do Rio Grande do Sul, Porto Alegre, Brazil; 2 Universidade Federal do Rio Grande do Sul, Porto Alegre, Brazil; 3 School of Medicine, Universidade Federal do Rio Grande do Sul Hospital de Clínicas de Porto Alegre, Rua Ramiro Barcelos, 2350 Fisiatria , Porto Alegre, RS 90035-903 Brazil

**Keywords:** Long COVID, Atherosclerosis, Endothelial dysfunction, Exercise training, Cardiovascular risk

## Abstract

**Purpose of Review:**

Post-COVID syndrome (PASC) has emerged as a multisystem condition associated with persistent cardiovascular abnormalities, including endothelial dysfunction, arterial stiffness, chronic inflammation, metabolic disturbances, autonomic imbalance, and increased atherosclerotic risk. This review summarizes the current evidence regarding the pathophysiological mechanisms linking PASC to cardiovascular disease and discusses the potential role of exercise training as a strategy to mitigate vascular dysfunction and cardiovascular risk.

**Recent Findings:**

Recent studies demonstrate that individuals with PASC exhibit persistent low-grade inflammation, impaired endothelial function, accelerated vascular aging, platelet hyperreactivity, insulin resistance, sarcopenia, and autonomic dysfunction. These alterations contribute to a pro-atherogenic phenotype that may persist months to years after SARS-CoV-2 infection. Emerging evidence indicates that exercise training improves inflammatory status, endothelial function, arterial stiffness, metabolic health, autonomic regulation, and functional capacity in post-COVID patients, potentially attenuating mechanisms involved in atherosclerotic progression.

**Summary:**

Post-COVID syndrome is associated with multiple interconnected biological pathways that increase long-term cardiovascular risk. Exercise training appears to be a promising non-pharmacological intervention capable of targeting several of these mechanisms simultaneously. Although further randomized controlled trials are needed, current evidence supports the integration of individualized exercise-based rehabilitation into the management of patients with PASC to promote vascular recovery and reduce cardiovascular risk.

**Graphical Abstract:**

**Figure:**
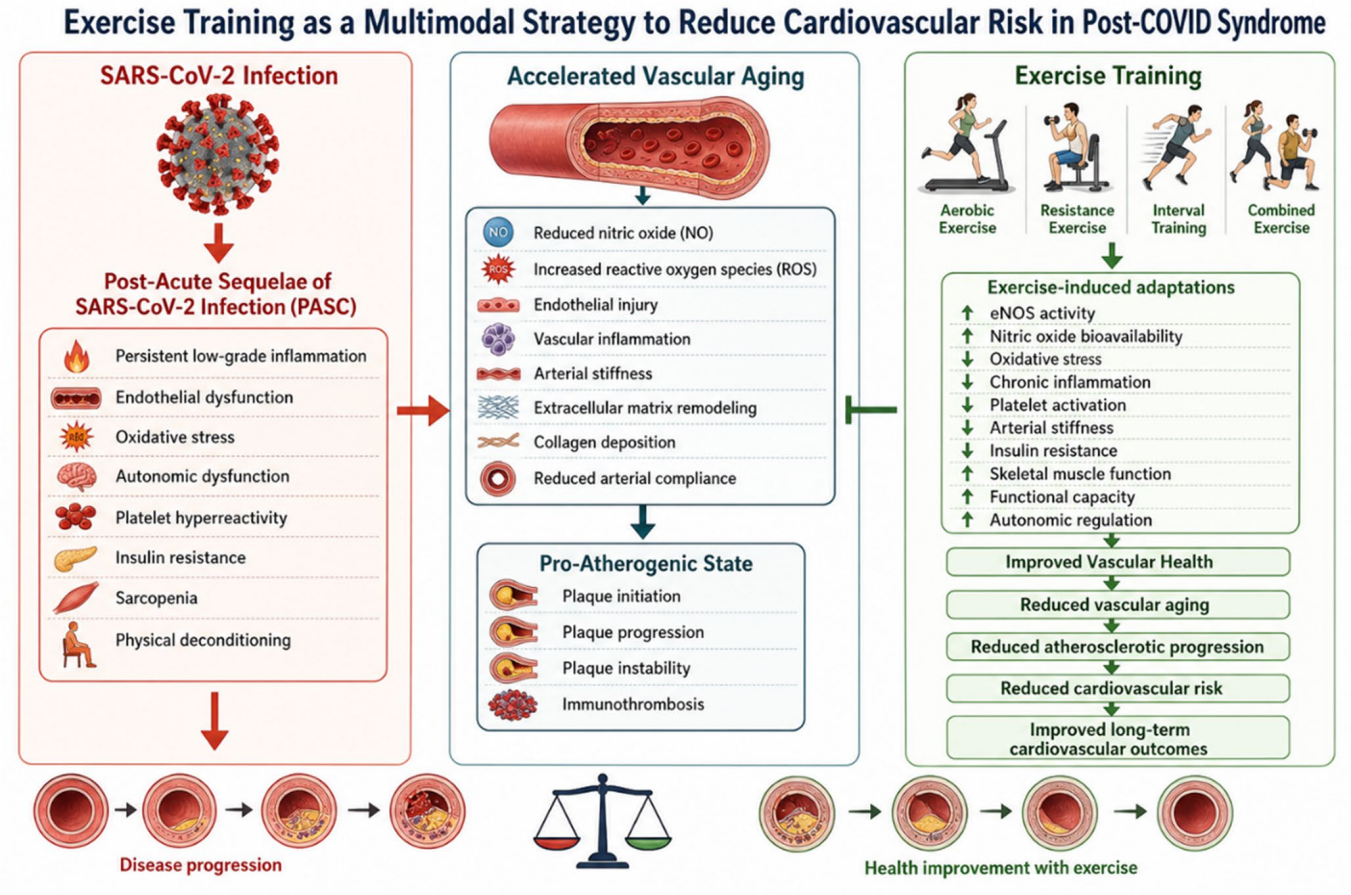

## Introduction

Post-COVID syndrome, also known as post-acute sequelae of severe acute respiratory syndrome coronavirus 2 (SARS-CoV-2) infection (PASC), is a complex multisystem condition characterized by persistent symptoms [1]. The condition is defined as the development or persistence of clinical sequelae after 4 weeks of an acute COVID-19 infection, with symptoms frequently extending beyond 12 months in a substantial proportion of survivors [1]. While initial clinical concerns primarily focused on respiratory complications and pulmonary sequelae, accumulating epidemiological and mechanistic evidence highlights a significant and persistent increase in long-term cardiovascular risk among COVID-19 survivors, regardless of disease severity during acute infection [1–4].

This elevated cardiovascular risk extends far beyond those who experienced severe disease requiring hospitalization, affecting even individuals with mild initial symptoms who recovered without requiring intensive care [2, 4, 5]. COVID-19 survivors maintain a elevated risk for myocardial infarction, ischemic stroke, heart failure, and cardiovascular death for at least 12 months following acute infection, with some evidence suggesting persistence of elevated risk beyond this timeframe [3, 4].

The CARTESIAN study [6], a large multicenter investigation, demonstrated that COVID-19 infection can increase arterial stiffness markers by approximately 5 years of vascular aging, supporting the concept of accelerated biological vascular aging [6, 7]. These structural and functional vascular alterations create a highly pro-atherogenic environment, characterized by enhanced lipid accumulation, plaque formation, and plaque instability, thereby predisposing patients to premature atherosclerosis and adverse cardiovascular events [6, 7].

Physical exercise, widely recognized for its pleiotropic cardiovascular benefits and anti-inflammatory properties, emerges as a potential non-pharmacological strategy to mitigate cardiovascular risk [8, 9]. Regular physical activity exerts potent anti-inflammatory, endothelial-protective, and metabolic-regulating effects, directly counteracting the pro-atherogenic mechanisms driven by SARS-CoV-2 infection [10, 11].

However, whether regular physical exercise can specifically reverse SARS-CoV-2-induced vascular aging and reduce atherosclerotic risk in PASC remains incompletely defined, and no prior review has systematically integrated mechanistic, epidemiological, and interventional evidence on this topic.

Therefore, the objective of this article is to describe the pro-atherogenic mechanisms of PASC, with emphasis on accelerated vascular aging and endothelial dysfunction. We then critically evaluate the evidence for physical exercise as an intervention to mitigate cardiovascular risk and restore vascular health in this population.

## Methods

This narrative review was conducted through a comprehensive and structured search of the scientific literature to identify the most relevant evidence regarding the relationship between post-COVID syndrome, cardiovascular risk, vascular dysfunction, and exercise training. Electronic searches were performed in PubMed/MEDLINE, Embase, Scopus, and Web of Science, covering publications from January 2020 through March 2026. The search strategy combined Medical Subject Headings (MeSH) and free-text terms related to post-COVID syndrome, cardiovascular complications, vascular biology, and exercise rehabilitation. The principal search terms included “Post-Acute Sequelae of SARS-CoV-2”, “Long COVID”, “PASC”, “COVID-19”, “exercise”, “exercise training”, “physical activity”, “cardiac rehabilitation”, “vascular aging”, “endothelial dysfunction”, “flow-mediated dilation”, “arterial stiffness”, “pulse wave velocity”, “atherosclerosis”, “vascular remodeling”, “autonomic dysfunction”, “cardiovascular risk”, “inflammation”, and “oxidative stress”. Boolean operators (“AND” and “OR”) were applied to optimize retrieval of relevant studies.

Original investigations, randomized controlled trials, prospective and retrospective cohort studies, case-control studies, systematic reviews, meta-analyses, narrative reviews, and experimental studies published in English were considered eligible when they provided clinically or mechanistically relevant information addressing cardiovascular alterations following SARS-CoV-2 infection or the effects of exercise interventions in individuals with post-COVID syndrome. Reference lists of all selected articles were manually examined to identify additional relevant publications that were not retrieved during the initial electronic search.

Studies focusing exclusively on acute COVID-19 without evaluation of persistent cardiovascular or vascular consequences, publications lacking peer review, conference abstracts without full manuscripts, editorials without original scientific data, and articles unrelated to cardiovascular pathophysiology or exercise interventions were excluded. Because the objective of the present manuscript was to provide a comprehensive and clinically oriented synthesis of the available evidence rather than to quantitatively estimate treatment effects, a formal systematic review protocol or meta-analytic methodology was not employed.

Emphasis was placed on recent advances published during 2024–2026, reflecting the rapidly evolving understanding of post-COVID cardiovascular complications. The evidence was organized into major pathophysiological domains, including persistent inflammation, endothelial dysfunction, arterial stiffness, vascular remodeling, platelet activation, metabolic dysregulation, autonomic dysfunction, and physical deconditioning, followed by a critical appraisal of the current evidence supporting exercise training as a potential strategy to mitigate these abnormalities. Whenever available, evidence derived from randomized controlled trials and systematic reviews was prioritized over observational studies to provide the highest level of evidence supporting clinical recommendations.

### Post-COVID Syndrome as a Pro-Atherogenic

#### Persistent Low-Grade Inflammation

Observational and cohort studies consistently demonstrate sustained elevations in inflammatory markers, particularly C-reactive protein (CRP), neutrophil counts, and composite indices such as the neutrophil-to-lymphocyte ratio, months after the acute phase [12]. In a large retrospective cohort of over 4,000 patients, PASC symptoms were significantly associated with CRP and peripheral inflammatory cell profiles, suggesting an ongoing inflammatory milieu beyond viral clearance [12]. Similarly, data from the multinational ORCHESTRA cohort revealed CRP levels within the range of low-grade inflammation at 12–18 months post-infection, reinforcing the chronic nature of this response [13]. Cross-sectional analyses further corroborate these findings, linking subtle but sustained increases in CRP, fibrinogen, and leukocyte-derived indices with PASC manifestations [14].

This inflammatory state is likely driven by sustained immune activation, potentially triggered by damage-associated molecular patterns and residual immune dysregulation [14, 15]. Key cytokines such as interleukin-6 (IL-6) and tumor necrosis factor-alpha (TNF-α) play central roles in maintaining this pro-inflammatory environment by promoting endothelial activation and leukocyte recruitment [16, 17]. Chronic inflammation is a well-established driver of atherosclerosis, contributing to endothelial dysfunction, oxidative stress, and plaque progression [17, 18].

**Fig. 1:**
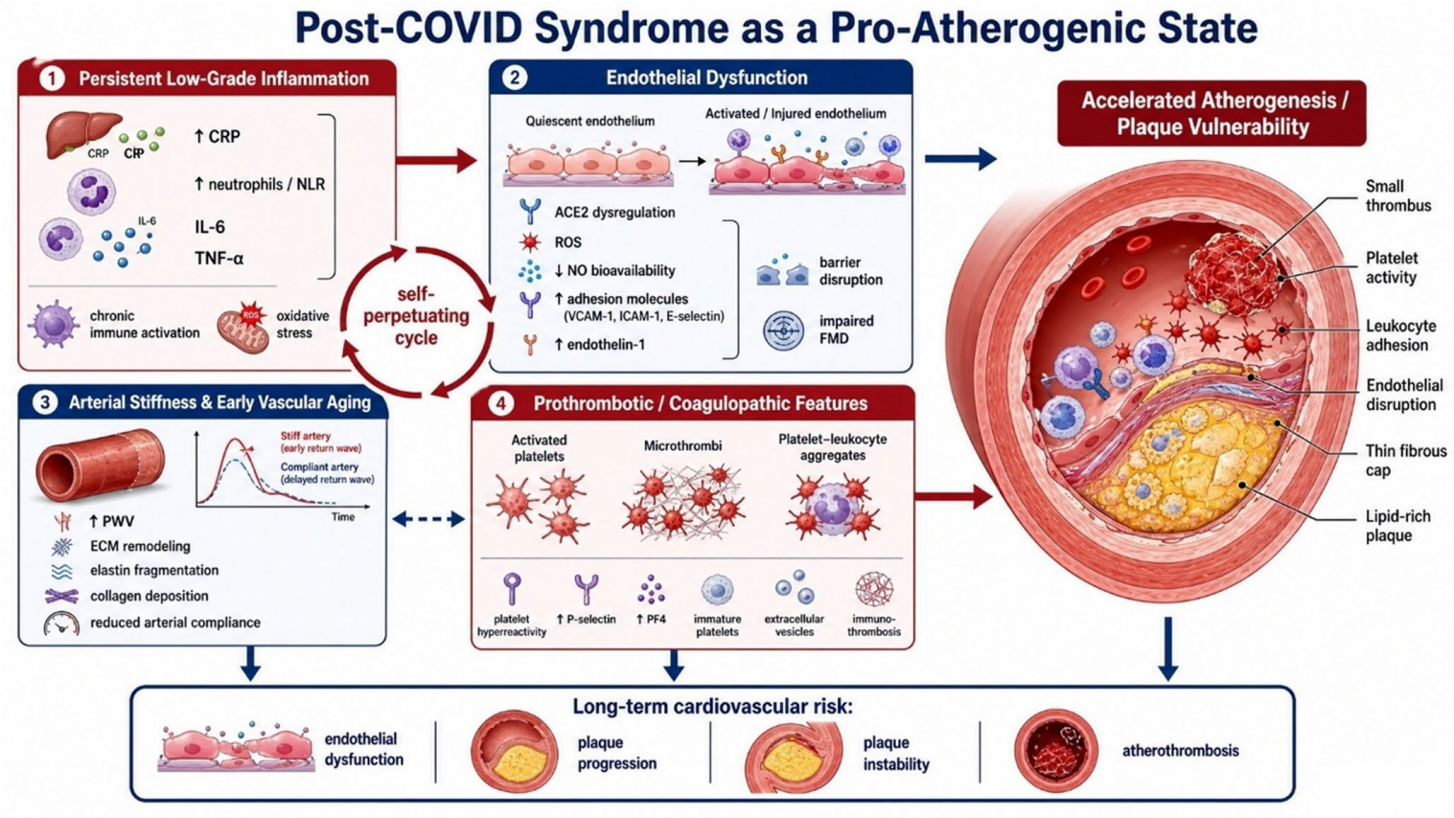
Post-COVID syndrome represents a pro-atherogenic state. Persistent low-grade inflammation following SARS-CoV-2 infection, characterized by elevated inflammatory biomarkers (e.g., C-reactive protein [CRP], interleukin-6 [IL-6], tumor necrosis factor-alpha [TNF-α]), and an increased neutrophil-to-lymphocyte ratio [NLR], contributes to chronic immune activation and oxidative stress. These mechanisms promote endothelial dysfunction through ACE2 dysregulation, increased reactive oxygen species (ROS) production, reduced nitric oxide (NO) bioavailability, enhanced expression of endothelial adhesion molecules, and elevated endothelin-1 levels, resulting in impaired vascular function and barrier disruption. Concurrently, a prothrombotic milieu develops, characterized by platelet hyperreactivity, microthrombi formation, platelet–leukocyte aggregates, extracellular vesicle release, and immunothrombosis. Together, these alterations accelerate arterial stiffness, extracellular matrix remodeling, elastin fragmentation, collagen deposition, and vascular aging. The interaction among inflammation, endothelial dysfunction, vascular remodeling, and coagulation abnormalities establishes a self-perpetuating cycle that promotes atherosclerotic plaque development, progression, and vulnerability. These processes increase the risk of plaque instability, atherothrombosis, and long-term cardiovascular complications in individuals with post-COVID syndrome. Figure are original and created by the authors using BioRender.com. Abbreviations: *ACE2* angiotensin-converting enzyme 2, *CRP*, C-reactive protein, *IL-6* interleukin-6, *NLR* neutrophil-to-lymphocyte ratio, *NO* nitric oxide, *ROS* reactive oxygen species, *TNF-α* tumor necrosis factor-alpha

Figure 1 summarizes the proposed pathophysiological pathways linking persistent inflammation, endothelial injury, vascular aging, and coagulation abnormalities to accelerated atherosclerosis and plaque vulnerability in individuals with Post-COVID Syndrome.

#### Endothelial Dysfunction

Endothelial dysfunction is a key mechanism linking PASC to long-term vascular complications. SARS-CoV-2 disrupts endothelial homeostasis through both direct viral effects and indirect inflammatory pathways, leading to endothelial activation, apoptosis, and barrier dysfunction [19–21]. Viral interaction with angiotensin-converting enzyme 2 (ACE2) promotes receptor internalization and dysregulation of the renin–angiotensin system, shifting vascular balance toward vasoconstriction, inflammation, and pro-thrombotic signaling [21, 22].

Beyond direct infection, experimental data suggest that the spike protein alone can induce vascular injury. In vitro studies demonstrate increased reactive oxygen species production, activation of hypoxia-inducible pathways, and disruption of endothelial barrier integrity following spike protein exposure [21, 23]. These alterations are accompanied by upregulation of vascular endothelial growth factor and inflammatory mediators, reinforcing a sustained dysfunctional vascular phenotype [23, 24]. However, the relative contribution of direct viral endothelial infection versus systemic inflammation remains incompletely established.

Clinically, endothelial dysfunction is characterized by increased expression of adhesion molecules, elevated circulating endothelial cells, and higher levels of vasoconstrictive mediators such as endothelin-1, reflecting a shift toward a procoagulant state [25]. This phenotype is further supported by impaired nitric oxide bioavailability, a hallmark of vascular dysfunction. Experimental and clinical evidence indicates that COVID-19 is associated with reduced endothelial nitric oxide synthase activity and lower nitric oxide levels, partly driven by oxidative stress and endothelial injury [23–25]. In line with these mechanisms, functional vascular assessments consistently demonstrate impaired endothelium-dependent vasodilation, as reflected by reduced flow-mediated dilation (FMD) in both acute and post-COVID patients [26, 27]. As FMD is largely nitric oxide-mediated, these findings provide translational evidence linking molecular changes to clinically measurable vascular dysfunction [26].

#### Arterial Stiffness and Early Vascular Aging

Arterial stiffness represents a key downstream consequence of COVID-19–related vascular injury, reflecting the cumulative effects of inflammation and endothelial dysfunction. Pulse wave velocity (PWV), the gold-standard marker of large artery stiffness, is consistently elevated across different clinical settings in COVID-19 patients. A meta-analysis by Jannasz et al. [28] demonstrated significantly higher carotid–femoral PWV in infected individuals compared to controls, supporting a direct association between SARS-CoV-2 infection and increased arterial stiffness. Similarly, Schnaubelt et al. [29] reported elevated PWV values in hospitalized patients, independently associated with COVID-19 status and correlated with worse clinical outcomes, including longer hospital stay and mortality.

Beyond the acute phase, arterial stiffness appears to carry prognostic and long-term implications. Stamatelopoulos et al. [30] showed that estimated PWV improves risk stratification for all-cause mortality, providing incremental predictive value beyond traditional risk factors. Longitudinal data from the multicenter CARTESIAN study further demonstrated persistently increased PWV up to 6 months after infection, supporting the concept of accelerated vascular aging following COVID-19 [31]. These findings are reinforced by imaging-based studies demonstrating increased aortic stiffness and altered wall shear stress, indicative of structural vascular remodeling [30].

These alterations reflect complex vascular remodeling processes driven by persistent inflammation and endothelial dysfunction, including extracellular matrix reorganization, elastin fragmentation, increased collagen deposition, and changes in wall shear stress, all of which contribute to reduced arterial compliance [30]. Importantly, increased arterial stiffness is not only a marker of vascular aging but also a mediator of atherosclerosis progression and plaque instability. This remodeling phenotype promotes early vascular aging and may accelerate atherosclerosis and increase long-term cardiovascular risk.

#### Prothrombotic and Coagulopathic Features

A hallmark of COVID-19–related coagulopathy is marked platelet hyperreactivity, which appears to persist beyond the acute phase and may contribute to long-term vascular risk. Studies consistently demonstrate that circulating platelets from COVID-19 patients exhibit a lower activation threshold and exaggerated responses to physiological agonists. Platelets from infected patients show markedly increased dense granule secretion and ADP release, with up to 30–90-fold increases in agonist-induced responses, alongside elevated levels of platelet activation markers such as P-selectin and platelet factor 4.^28^ Similarly, enhanced aggregation, spreading, and platelet–leukocyte aggregate formation further support this hyperreactive phenotype, partly mediated by MAPK pathway activation and increased thromboxane generation [32, 33].

This phenotype is further reinforced by increased platelet turnover, with increased levels of immature platelets, which are highly reactive and associated with disease severity,^30^ as well as sustained platelet activation and procoagulant extracellular vesicle release months after infection. Platelet hyperreactivity correlates with inflammatory burden and clinical severity, highlighting the interplay between thromboinflammation and disease progression [33, 34].

Beyond thrombosis, activated platelets may directly contribute to plaque instability. Platelet-derived mediators promote endothelial activation, leukocyte recruitment, and local inflammatory amplification, fostering a prothrombotic and pro-atherogenic microenvironment [33, 34]. Collectively, these mechanisms support a persistent procoagulant state in PASC, linking platelet dysregulation to atherothrombosis and increased cardiovascular risk.

#### Metabolic Disturbances and Physical Deconditioning

Metabolic disturbances and physical deconditioning are interrelated components of PASC, contributing to persistent symptoms and increased cardiometabolic risk. Insulin resistance has emerged as a central feature, affecting even individuals without prior metabolic disease [35, 36]. Longitudinal data demonstrate sustained abnormalities in glucose homeostasis, including persistent insulin resistance and new-onset diabetes up to 12 months after infection. This disruption is closely linked to chronic inflammation, which independently predicts worsening insulin sensitivity in post-COVID patients [35, 36]. Mechanistically, alterations in insulin signaling pathways, endothelial dysfunction, and dysregulation of metabolic mediators further contribute to impaired glucose metabolism [37].

In parallel, sarcopenia has been consistently identified as a highly prevalent and persistent consequence of COVID-19. A meta-analysis including 39 studies, and 6,982 individuals demonstrated that sarcopenia affects 48.7% of patients during the acute phase and remains present in 23.5% of long COVID patients[38, 39]. Interestingly, pooled data did not demonstrate significant associations with length of hospital stay, intensive care unit admission, mechanical ventilation, or mortality in acute COVID-19, although an increased likelihood of tracheostomy was observed [38]. Although these findings suggest that the short-term prognostic impact of sarcopenia remains uncertain, observational studies indicate that reduced muscle mass and function may still be associated with worse clinical trajectories in specific populations.

These changes are driven by hyperinflammation, catabolic stress, reduced nutritional intake, and prolonged immobilization, promoting sustained muscle loss and impaired recovery [39].

Physical deconditioning represents both a key downstream consequence and a perpetuating factor. Cardiopulmonary exercise testing studies show that reduced exercise capacity is frequently driven by deconditioning rather than primary cardiopulmonary limitations. Hospitalization, inactivity, and prolonged recovery contribute to persistent functional impairment despite rehabilitation [40, 41]. Importantly, deconditioning interacts with inflammation, microvascular dysfunction, and skeletal muscle abnormalities, forming a self-reinforcing cycle.

### Impact of Exercise on Inflammatory Risk in Post-COVID Syndrome

Regular physical exercise has emerged as a key non-pharmacological strategy to mitigate persistent inflammation in PASC, acting as a potent anti-inflammatory modulator through multiple systemic pathways. This effect is particularly relevant, given that post-COVID condition is frequently characterized by chronic low-grade inflammation (as discussed in previous sections), which contributes to persistent symptoms and elevated cardiovascular risk.

One of the primary mechanisms underlying this response involves the release of myokines – cytokines produced and secreted by skeletal muscle during contraction. Among these, irisin has gained attention due to its role in improving metabolic homeostasis and attenuating inflammatory signaling pathways. Exercise-induced irisin appears to downregulate pro-inflammatory cascades, including the MAPK and TLR4/MyD88 pathways, while enhancing anti-inflammatory responses, potentially counteracting the cytokine dysregulation observed in COVID-19 [42, 43].

In addition to myokines, exercise also modulates classical inflammatory biomarkers. Moderate-intensity exercise is associated with reductions in pro-inflammatory cytokines such as TNF-α and IL-6, alongside increases in anti-inflammatory mediators like IL-10. Importantly, the behavior of IL-6 in this context is complex: although elevated in chronic inflammation, exercise-induced IL-6 acts transiently as a myokine with anti-inflammatory properties, stimulating the production of IL-1 receptor antagonist and IL-10 [44]. Furthermore, structured exercise interventions consistently reduce systemic inflammatory markers, including CRP, reinforcing their role in attenuating low-grade inflammation.

Clinical evidence supports these mechanistic findings. In a network meta-analysis by Du et al., [44] comprising 33 randomized controlled trials and 2,895 participants, exercise interventions significantly improved functional capacity, dyspnea, and quality of life in individuals with PASC, with combined aerobic and respiratory training showing the greatest benefits.

Direct evidence on inflammatory biomarkers is provided by a randomized controlled trial conducted by Tartibian et al. [45] involving 296 COVID-19 survivors (aged 20–93 years), in which 8 weeks of moderate-intensity continuous training, resistance training, or combined exercise resulted in significant reductions in CRP levels, along with improvements in multiple hematological and biochemical markers compared to a non-exercise group. Notably, combined exercise training yielded superior effects, suggesting a synergistic anti-inflammatory benefit of multimodal interventions.

More specific immunological modulation has also been demonstrated in smaller mechanistic studies. In a prospective clinical trial involving 24 patients recovering from moderate to severe COVID-19, a 12-week supervised exercise program led to significant reductions in key pro-inflammatory chemokines, including CXCL8/IL-8 and CCL2/MCP-1, as well as decreased IFN-γ levels [46]. These changes were not observed in the unsupervised home-based group, highlighting supervision and program structure as key determinants of anti-inflammatory efficacy.

Complementary evidence comes from observational data. In a cohort of 227 post-COVID patients evaluated 6–12 months after hospitalization, de Castro et al. [47] reported sustained alterations in cytokine profiles associated with reduced physical function. This finding underscores the close interplay between inflammation and functional impairment in PASC, supporting the rationale for interventions targeting both domains.

**Fig. 2:**
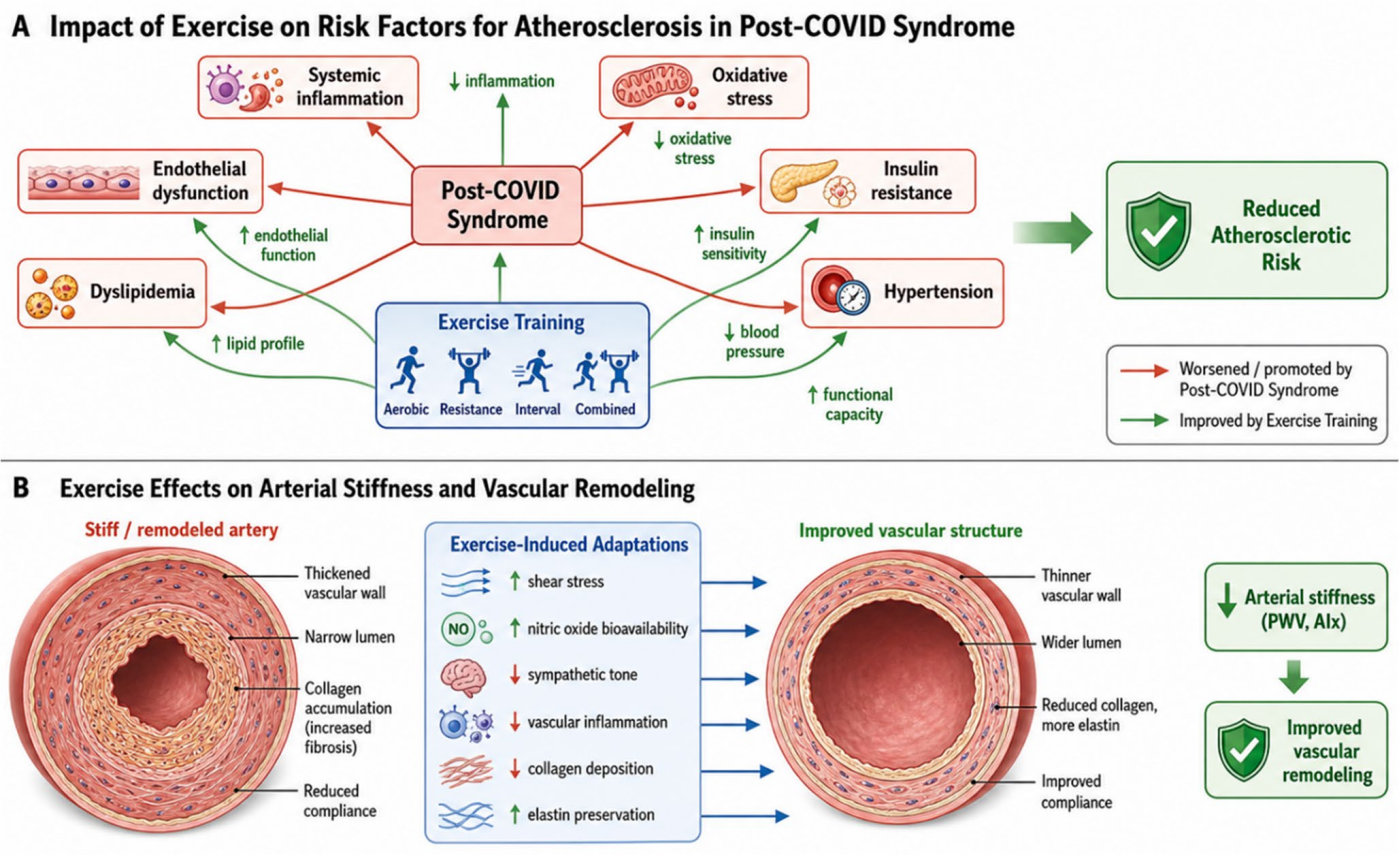
The potential effects of exercise training on atherosclerotic risk and vascular remodeling in post-COVID syndrome. (**A**) Post-COVID syndrome is associated with multiple cardiovascular risk factors that contribute to atherosclerosis, including systemic inflammation, oxidative stress, endothelial dysfunction, dyslipidemia, insulin resistance, and hypertension. Exercise training—including aerobic, resistance, interval, and combined modalities—may counteract these pathophysiological alterations by reducing inflammation and oxidative stress, improving endothelial function, enhancing lipid profile and insulin sensitivity, lowering blood pressure, and increasing functional capacity. Through these mechanisms, regular exercise may attenuate the pro-atherogenic effects of post-COVID syndrome and reduce the overall atherosclerotic risk. Red arrows indicate processes promoted or worsened by post-COVID syndrome, whereas green arrows indicate pathways improved by exercise training. (**B**) Proposed mechanisms by which exercise training improves arterial stiffness and vascular remodeling in individuals with post-COVID syndrome. A stiffened and remodeled artery is characterized by vascular wall thickening, lumen narrowing, collagen accumulation (fibrosis), and reduced arterial compliance. Exercise-induced adaptations—including increased shear stress, enhanced nitric oxide (NO) bioavailability, reduced sympathetic activity, decreased vascular inflammation, reduced collagen deposition, and preservation of elastin fibers—promote favorable structural and functional vascular changes. These adaptations result in thinner vascular walls, wider arterial lumen, reduced fibrosis, improved arterial compliance, and lower arterial stiffness, reflected by reductions in pulse wave velocity (PWV) and augmentation index (AIx). Collectively, these effects contribute to improved vascular remodeling and cardiovascular health following COVID-19. Original figure created by the authors using BioRender.com. Abbreviations: *AIx* augmentation index, *NO* nitric oxide, *PWV* pulse wave velocity

From a cardiovascular perspective, the anti-inflammatory effects of exercise have important implications. Chronic inflammation promotes endothelial dysfunction, oxidative stress, and thromboinflammation – key drivers of atherosclerotic progression. Exercise mitigates these processes by reducing inflammatory mediators, improving nitric oxide bioavailability, and promoting vascular homeostasis [48]. Therefore, regular, moderate-intensity exercise may improve post-COVID-19 symptoms and reduce long-term cardiovascular risk in this population. Figure 2 summarizes the principal mechanisms through which exercise training may attenuate atherosclerosis-related risk factors and improve vascular structure and function in individuals with PASC.

### Exercise-Mediated Recovery of Endothelial Function

Exercise is a physiological and important strategy capable of restoring endothelial function through repeated increases in arterial blood flow and laminar shear stress. During continuous aerobic exercise, increased shear stress activates mechanosensitive endothelial pathways, including phosphorylation and upregulation of endothelial nitric oxide synthase (eNOS), thereby increasing nitric oxide (NO) bioavailability, considered the main mechanism of improvement in endothelial function mediated by aerobic exercise. NO promotes vasodilation, inhibits platelet aggregation and leukocyte adhesion, and supports an anti-inflammatory, antithrombotic endothelial phenotype [49]. These mechanisms are especially relevant after COVID-19, in which endothelial injury has been linked to reducing NO availability, oxidative stress, glycocalyx disruption, inflammation and microthrombotic signaling, which may lead to deep vein thrombosis, stroke, and microvascular complications through a pro-thrombotic mechanism [50].

Exercise training may also improve endothelial function by shifting the redox balance toward a more favorable vascular environment. Repeated bouts of aerobic exercise increase endogenous antioxidant capacity, reduce NADPH oxidase-related reactive oxygen species generation, and limit eNOS uncoupling [51]. Consequently, more NO remains available to vascular smooth muscle, improving endothelium-dependent vasodilation. This adaptive response is clinically captured by FMD, a non-invasive index of conduit artery endothelial function [51]. Robust evidence indicates that structured exercise interventions improve FMD across different clinical populations, with aerobic training being one of the most consistent modalities [52]. Exercise may improve the ability of arteries to respond appropriately to changes in flow demand, which is central to vascular health in patients recovering from systemic inflammatory insults such as COVID-19 [53].

In this scenario, post-COVID patients, impaired FMD has been associated with lower exercise capacity and worse ventilatory efficiency during CPET suggesting that endothelial dysfunction may contribute not only to vascular risk but also to persistent functional limitation [54]. Although direct trials testing exercise-induced endothelial recovery in long COVID remains limited, the biological plausibility is that exercise targets several mechanisms implicated in post-viral vascular injury, including low-grade inflammation, oxidative stress, impaired NO signaling or even physical inactivity, for example. This is relevant because physical inactivity itself can worsen endothelial function, creating a self-perpetuating cycle of symptoms, deconditioning, and vascular impairment [55]. Therefore, individualized aerobic rehabilitation may represent a vascular-directed therapy capable of partially reversing endothelial dysfunction after COVID-19, especially when initiated progressively and monitored according to symptoms, autonomic responses, and exercise tolerance.

### Exercise Effects on Arterial Stiffness and Vascular Remodeling

Arterial stiffness reflects the combined influence of vascular smooth muscle tone, blood pressure, extracellular matrix composition, and chronic inflammatory signaling. Pulse wave velocity (PWV), particularly carotid–femoral PWV, is the most widely used clinical marker of large elastic artery stiffness and is strongly associated with vascular aging and cardiovascular events [46]. After COVID-19, increased PWV has been proposed as a manifestation of accelerated vascular aging, potentially driven by endothelial injury, persistent inflammation, autonomic imbalance, and reduced physical activity [31].

Exercise training reduces arterial stiffness through both functional and structural mechanisms. Acute effects are predominantly functional, including reduced sympathetic vasoconstrictor tone, improved endothelial nitric oxide (NO) signaling, lower blood pressure, and enhanced vascular smooth muscle relaxation. These adaptations may decrease wave reflection and central pulsatile load even before measurable wall remodeling occurs [53]. In contrast, chronic exercise training induces structural changes, such as reduced collagen cross-linking, improved elastin-to-collagen balance, diminished vascular calcification signaling, and attenuation of inflammatory remodeling within the arterial wall. Thus, exercise may modulate both the dynamic regulation of arterial tone and the slower biological processes underlying vascular remodeling [41].

Aerobic exercise reduces PWV in adults with elevated cardiovascular risk, including hypertensive, prehypertensive, overweight, obese, and postmenopausal populations. The effect is more consistent when training lasts at least 12 weeks, at moderate intensity (e.g., 40–60% heart rate reserve), with regular weekly frequency and sufficient total volume (e.g., ≥ 150 min/week) [43]. These findings are particularly relevant to post-COVID conditions, as the vascular phenotype observed after infection resembles an inflammatory and accelerated aging state rather than an isolated respiratory sequela. However, data on high-intensity interval training remain less consistent in this context.

Extrapolating these findings to long COVID requires caution. Most available exercise trials were conducted in traditional cardiometabolic populations, not specifically in patients with post-viral symptoms, dysautonomia, or post-exertional symptom exacerbation [42]. Therefore, individualized exercise should be prescribed progressively, with careful monitoring of symptom response and recovery kinetics (e.g., heart rate recovery and symptom resolution within 24–48 h). Reductions in PWV may represent an intermediate vascular endpoint linking rehabilitation to reduced pulsatile stress, improved ventricular–arterial coupling (i.e., the efficiency of energy transfer from the left ventricle to the aorta), and potentially slower progression of atherosclerotic remodeling after COVID-19. Incorporating PWV alongside flow-mediated dilation (FMD) may help distinguish functional endothelial recovery from broader changes in arterial wall mechanics.

Table 1 summarizes the main vascular domains affected by exercise training, endothelial function, arterial stiffness, and vascular remodeling—alongside the proposed biological mechanisms, expected vascular adaptations, and their potential relevance to post-COVID atherosclerotic risk. Future studies should evaluate whether exercise-induced PWV reductions translate into lower cardiovascular event rates in post-COVID patients and whether baseline PWV can guide personalized rehabilitation strategies.

**Table 1:** Effects of exercise training on endothelial function, arterial stiffness, and vascular remodeling after COVID-19

Vascular domain	Exercise-related stimulus	Main biological target	Expected vascular effect	Relevance after COVID-19
Endothelial function [19, 21, 22, 56, 57]	Repeated laminar shear stress during aerobic exercise	eNOS activation and NO bioavailability	Improved endothelium-dependent vasodilation and FMD	Potential recovery of impaired endothelial function
Oxidative balance [53, 57]	Regular aerobic training	Reduced ROS generation and improved antioxidant defenses	Less NO degradation and lower endothelial injury	May counteract post-inflammatory oxidative stress
Vascular inflammation [42, 53]	Repeated moderate-intensity exercise exposure	Lower cytokine signaling and leukocyte adhesion	Anti-inflammatory endothelial phenotype	May reduce persistent vascular activation
Arterial stiffness [28, 43]	Reduced blood pressure and sympathetic tone	Vascular smooth muscle relaxation	Lower PWV and wave reflection	May attenuate accelerated vascular aging
Vascular remodeling [6]	Long-term training adaptation	Extracellular matrix, collagen–elastin balance, calcification signaling	Slower structural stiffening	Potential protection against atherosclerotic progression

eNOS - endothelial nitric oxide synthase; NO - nitric oxide; FMD - flow-mediated dilation; ROS - reactive oxygen species; PWV - pulse wave velocity

### Exercise and Metabolic Atherosclerotic Risk Factors in Post-COVID Patients

COVID-19 is a multisystemic disease capable of inducing a persistent cardiometabolic syndrome characterized by low-grade inflammation, insulin resistance, dyslipidemia, and autonomic dysfunction—factors that significantly amplify atherosclerotic risk during the post-acute period (> 4 weeks after infection) [58]. Physical exercise emerges as a key therapeutic intervention to modulate these metabolic risks and restore cardiovascular function [58].

Exercise prescription for patients with Post-Acute Sequelae of SARS-CoV-2 (PASC) should be multidisciplinary, individualized, and progressively structured, aiming to reverse muscle atrophy and restore metabolic and cardiovascular function [59, 60]. Due to persistent symptoms such as chronic fatigue and effort intolerance, physical rehabilitation is considered the central pillar for functional recovery [61, 62]. Training programs that integrate strength and aerobic exercises (combined training) are widely recommended, showing superior effects compared to isolated modalities by simultaneously improving cardiorespiratory fitness and muscle function [63].

Early exercise-based rehabilitation is recommended to decrease neuromuscular decline and accelerate recovery of mitochondrial function and fat oxidation [60, 62]. Programs with an average duration of 12 weeks show the best metabolic outcomes [63, 64]. Training frequency typically ranges from 3 to 6 sessions per week, depending on the setting (e.g., hospital-based or home-based), with lower frequencies often better tolerated in early stages [61, 63].

The aerobic component is typically prescribed at intensities of 50% to 80% of peak VO₂ or 60% to 75% of heart rate reserve, with session durations of up to 90 min [61, 63]. For resistance exercise, intensity ranges between 70% and 85% of one-repetition maximum (1RM), with 3 sets of 8 to 12 repetitions for major muscle groups [61, 63]. Lower starting intensities are recommended for deconditioned patients.

In patients with severe fatigue or pulmonary damage, structured exercise should focus on respiratory efficiency, with breathing exercises associated with training [61]. Load should be adjusted according to individual capacity [59, 60], with close attention to pre-exercise screening. Exercise is relatively contraindicated in patients with resting heart rate > 100 bpm [59], oxygen saturation < 95% [59, 61], or systolic blood pressure < 90 mmHg or > 140 mmHg [59]. Post-exertional symptom exacerbation should be monitored for 24–48 h post-exercise.

### Exercise and Autonomic Regulation as an Indirect Modifier of Atherosclerotic Risk

The autonomic nervous system (ANS) undergoes significant alterations in post-COVID syndrome, a phenomenon frequently described as dysautonomia [59, 60, 62, 65]. Dysautonomia in PASC is recognized as a central pathophysiological mechanism that mimics conditions such as Postural Orthostatic Tachycardia Syndrome (POTS) and chronic fatigue syndrome. Clinically, it manifests through palpitations, chest pain, and orthostatic intolerance [59, 65], affecting up to 30–40% of patients and compromising the homeostasis of multiple organ systems [65].

Analysis of Heart Rate Variability (HRV) and sympathovagal balance reveals that the autonomic nervous system exerts a primary role over cardiac function. A reduction in HRV—driven by sympathetic overactivity and vagal withdrawal—is frequently associated with adverse prognosis and arrhythmic risk [66]. These factors are further linked to endothelial dysfunction and increased systemic inflammation, both of which contribute to overall atherosclerotic risk [67]. HRV assessment offers a non-invasive, clinically accessible tool to monitor autonomic function in this population.

There is a direct relationship between sympathetic hyperactivity and the progression of atherosclerosis, as continuous adrenergic stress and systemic inflammation induce damage to cardiac structure and electrophysiology, promoting adverse remodeling [66, 68, 69]. Persistent endothelial dysfunction in the post-COVID period favors a pro-atherosclerotic and pro-thrombotic phenotype, accelerating plaque instability and increasing the risk of acute coronary events [68]. The synergistic interaction between inflammation and sympathetic stress exacerbates the imbalance between myocardial oxygen supply and demand [69], potentially explaining the increased incidence of cardiovascular events reported in post-COVID cohorts.

Given these mechanisms, exercise training emerges as a promising strategy to counteract autonomic dysfunction and its vasculares consequences. Exercise acts as an important indirect modulator of atherosclerotic risk by reducing peripheral arterial resistance and vascular tone [62, 63, 66]. Parasympathetic reactivation during the recovery phase assists in stabilizing ventricular repolarization [66]. Exercise also reduces resting heart rate and restores mitochondrial function, effectively combating the chronotropic intolerance associated with PASC [62]. These adaptations promote a significant reduction in systolic blood pressure, which may ultimately slow the progression of atherosclerosis [63]. Moderate-intensity aerobic training appears particularly effective for enhancing parasympathetic tone and improving HRV.

The available randomized evidence on exercise training in post-COVID syndrome is summarized in Table 2. Overall, randomized trials suggest that structured exercise rehabilitation improves functional capacity, muscle strength, fatigue, dyspnea and, in some studies, quality of life. However, most trials were limited by small sample sizes, short follow-up, heterogeneous exercise prescriptions and limited assessment of vascular endpoints. Therefore, although current evidence supports exercise-based rehabilitation as a promising strategy for functional recovery in PASC, its direct effects on endothelial function, arterial stiffness, vascular remodeling and long-term cardiovascular outcomes remain insufficiently established.

**Table 2:** Randomized controlled trials and systematic reviews/meta-analyses evaluating exercise training in individuals with post-COVID syndrome

Study	Design and population	Intervention	Duration	Outcomes	Main findings	Relevance
Tartibian et al., [45]	Randomized controlled assessor-blinded trial including COVID-19 survivors aged 20–93 years	Moderate-intensity continuous training, resistance training, or combined exercise compared with non-exercise control	8 weeks	Biochemical and hematological markers, including CRP and systemic recovery markers	Exercise training improved several biochemical and hematological outcomes. Combined exercise appeared to provide the most consistent benefits, including reductions in inflammatory status	Provides direct randomized evidence that structured exercise may attenuate persistent systemic inflammation after COVID-19
Sick et al., [70]	Parallel-group randomized controlled trial in adults with post-COVID syndrome who had symptoms for more than 12 weeks and did not require hospitalization during acute infection	Supervised endurance training, concurrent training, or non-exercising control	12 weeks	VO₂peak, muscle strength, heart rate variability, fatigue, symptoms, health-related quality of life and concentration performance	Both endurance and concurrent training increased VO₂peak and reduced fatigue. Concurrent training produced greater improvements in breathlessness and lower-body strength.	Supports the feasibility and safety of supervised exercise rehabilitation and reinforces the role of aerobic and combined training in improving functional recovery
Randomized trials summarized by Ferrari et al., [11]	Systematic review and meta-analysis of randomized controlled trials including adults with post-COVID syndrome	Aerobic training, high-intensity interval training, combined aerobic plus resistance training, and usual care comparisons	≥ 4 weeks	VO₂peak, six-minute walk distance, muscle strength, quality of life, anxiety, depression, VE/VCO₂ slope, OUES and O₂ pulse	Eighteen RCTs including 1,171 participants were analyzed. Exercise training improved functional capacity and muscle strength, although certainty of evidence was low to very low. HIIT showed the largest VO₂peak gain, while effects on quality of life and cardiopulmonary efficiency outcomes were less consistent.	Provides the most comprehensive synthesis of randomized evidence on exercise modalities in post-COVID individuals and supports the need for individualized exercise prescription.
Randomized trials summarized by Du et al., [44]	Systematic review and network meta-analysis of exercise interventions for post-COVID syndrome	Aerobic training, respiratory training, resistance training and combined exercise approaches	Variable across included studies	Functional capacity, dyspnea, fatigue and quality of life	Exercise interventions improved functional capacity, dyspnea and quality of life, with combined aerobic and respiratory training showing particularly favorable effects in the network analysis.	Supports the clinical efficacy of exercise-based rehabilitation in PASC and reinforces the rationale for multimodal interventions.

*CRP* C-reactive protein, *FMD* flow-mediated dilation, *HIIT* high-intensity interval training, *OUES* oxygen uptake efficiency slope, *PASC* post-acute sequelae of SARS-CoV-2 infection, *PWV* pulse wave velocity, *RCT* randomized controlled trial, *VO₂peak* peak oxygen uptake

## Limitations

Despite the growing body of evidence linking post-COVID syndrome to persistent cardiovascular abnormalities, several limitations within the current literature should be acknowledged when interpreting the findings summarized in this review. First, this manuscript represents a narrative review and, although a structured literature search was performed, it was not conducted according to a predefined systematic review protocol. Consequently, study selection was based on scientific relevance and methodological quality rather than formal risk-of-bias assessment, which may introduce an element of selection bias inherent to narrative reviews.

A second important limitation concerns the considerable heterogeneity among published studies evaluating post-COVID syndrome. Differences in the definition of post-COVID syndrome, duration of follow-up, severity of the initial infection, vaccination status, viral variants, demographic characteristics, and pre-existing comorbidities substantially limit direct comparisons across studies. Furthermore, many investigations include mixed populations of hospitalized and non-hospitalized individuals or combine patients with widely different symptom profiles, making it difficult to determine whether the observed cardiovascular alterations are consistent across all post-COVID phenotypes.

The evidence supporting exercise-based rehabilitation also presents limitations. Although an increasing number of randomized controlled trials have been published, most studies enrolled relatively small samples, evaluated short intervention periods, and primarily focused on functional outcomes such as exercise capacity, dyspnea, fatigue, or quality of life.

Another limitation involves the heterogeneity of exercise interventions reported in the literature. Considerable variability exists regarding exercise modality, intensity, frequency, duration, supervision, progression criteria, and rehabilitation setting. These methodological differences hinder direct comparisons among studies and currently preclude the identification of an optimal exercise prescription for individuals with post-COVID syndrome.

Finally, many mechanistic pathways discussed throughout this review are supported by experimental models, translational investigations, or extrapolation from other cardiovascular diseases characterized by endothelial dysfunction and chronic inflammation. Although these mechanisms are biologically plausible and consistent with current understanding of vascular physiology, caution should be exercised when translating these findings directly into clinical recommendations until further disease-specific evidence becomes available.

These limitations highlight the need for multicenter studies with standardized diagnostic criteria, comprehensive vascular phenotyping, longer follow-up, and clinically meaningful cardiovascular outcomes. Nevertheless, despite these limitations, the consistency of findings across epidemiological, mechanistic, and interventional studies provides a strong rationale for considering post-COVID syndrome as a condition associated with persistent cardiovascular vulnerability and supports continued investigation of exercise training as a promising strategy to mitigate long-term cardiovascular risk.

## Knowledge Gaps

Despite the rapid expansion of research on post-COVID syndrome, substantial knowledge gaps remain regarding the mechanisms responsible for persistent cardiovascular dysfunction and the role of exercise training in modifying long-term cardiovascular risk.

Another important limitation of the current literature is the absence of validated biomarkers capable of identifying patients at greatest cardiovascular risk following COVID-19. Although flow-mediated dilation, pulse wave velocity, endothelial-derived biomarkers, inflammatory cytokines, circulating endothelial cells, and measures of autonomic function have demonstrated promising results in observational studies, none has yet been validated as a reliable prognostic tool for routine clinical practice. The development of integrated vascular phenotyping strategies combining functional, biochemical, and imaging biomarkers may substantially improve risk stratification and facilitate individualized therapeutic approaches.

Most randomized clinical trials have primarily evaluated improvements in exercise capacity, fatigue, dyspnea, or quality of life, whereas relatively few studies have investigated vascular outcomes such as endothelial function, arterial stiffness, vascular remodeling, myocardial function, or subclinical atherosclerosis. Similarly, the optimal exercise prescription for different clinical phenotypes of post-COVID syndrome remains unknown. Whether aerobic training, resistance exercise, high-intensity interval training, combined exercise, inspiratory muscle training, or multimodal rehabilitation provides superior cardiovascular benefits has not yet been established.

Patients with dysautonomia, postural orthostatic tachycardia syndrome, severe fatigue, or post-exertional symptom exacerbation represent another important knowledge gap. These individuals are frequently excluded from clinical trials despite constituting a substantial proportion of patients with post-COVID syndrome. Consequently, there is limited evidence regarding the safety, tolerability, and efficacy of exercise-based rehabilitation in these specific subgroups. Future investigations should specifically address these populations to establish individualized rehabilitation strategies that maximize clinical benefit while minimizing symptom exacerbation.

Additional uncertainties concern the influence of demographic and clinical characteristics on cardiovascular recovery. Sex-related differences, aging, obesity, diabetes mellitus, hypertension, pre-existing cardiovascular disease, vaccination status, viral variants, and reinfection may all influence the persistence of vascular dysfunction and the response to exercise training.

## Furutre Directions

The rapid expansion of research on post-COVID syndrome has considerably improved our understanding of its cardiovascular manifestations; however, several fundamental questions remain unanswered and should guide future investigations. Given the complexity and heterogeneity of post-COVID syndrome, future research should move beyond descriptive observational studies toward large prospective multicenter investigations capable of establishing causal relationships between persistent vascular abnormalities, exercise interventions, and long-term cardiovascular outcomes. Such studies will be essential to determine whether the vascular alterations observed after SARS-CoV-2 infection represent reversible physiological adaptations or permanent pathological remodeling that predisposes individuals to premature cardiovascular disease.

Randomized controlled trials should constitute the next major step in this field. Future exercise intervention studies should be adequately powered and designed to compare different exercise modalities, including aerobic, resistance, interval, combined, and multimodal rehabilitation programs. In addition to traditional functional outcomes, future trials should incorporate objective vascular endpoints, including endothelial function assessed by flow-mediated dilation, arterial stiffness measured by carotid-femoral pulse wave velocity, central hemodynamics, myocardial function evaluated by advanced echocardiography or cardiac magnetic resonance imaging, and circulating biomarkers of endothelial injury, inflammation, oxidative stress, and thrombosis. The integration of these vascular biomarkers with clinical outcomes would provide a more comprehensive understanding of the mechanisms through which exercise influences cardiovascular recovery following COVID-19.

Future investigations should also prioritize long-term follow-up. Most currently available studies evaluate exercise interventions over periods ranging from 8 to 16 weeks, leaving uncertainty regarding the durability of vascular adaptations and their relationship with clinically meaningful outcomes. Longitudinal studies extending beyond one year are needed to determine whether improvements in endothelial function, arterial stiffness, inflammatory status, autonomic regulation, and metabolic health are sustained over time and whether these physiological adaptations ultimately translate into reductions in major adverse cardiovascular events, hospitalization, and cardiovascular mortality.

An equally important priority involves improving the phenotypic characterization of post-COVID syndrome. Current evidence suggests that post-COVID syndrome encompasses multiple clinical phenotypes with distinct biological mechanisms, symptom profiles, and recovery trajectories. Future studies should therefore adopt precision medicine approaches capable of identifying subgroups of patients who may respond differently to rehabilitation interventions. Variables such as age, sex, obesity, pre-existing cardiovascular disease, diabetes mellitus, vaccination status, viral variant, reinfection history, and severity of the acute infection should be systematically incorporated into study design and statistical analyses to facilitate personalized therapeutic strategies.

Special attention should also be directed toward populations that remain underrepresented in the current literature. Patients with postural orthostatic tachycardia syndrome, severe autonomic dysfunction, post-exertional symptom exacerbation, chronic fatigue, or significant exercise intolerance represent a substantial proportion of individuals with post-COVID syndrome but are frequently excluded from clinical trials. Dedicated studies are necessary to establish safe and effective rehabilitation protocols for these patients and to determine whether conventional exercise prescriptions require modification according to individual physiological responses.

Technological innovations are expected to play an increasingly important role in future rehabilitation programs. Wearable devices capable of continuously monitoring heart rate, physical activity, oxygen saturation, heart rate variability, and recovery kinetics may facilitate individualized exercise prescription and early identification of symptom exacerbation. Furthermore, advances in artificial intelligence and machine learning offer opportunities to integrate clinical, physiological, imaging, and biomarker data into predictive models capable of identifying patients at greatest cardiovascular risk and optimizing personalized rehabilitation strategies.

Finally, future research should seek to integrate mechanistic discoveries with clinical implementation. Understanding how persistent inflammation, endothelial dysfunction, arterial stiffness, autonomic dysregulation, and metabolic abnormalities interact to influence cardiovascular prognosis may facilitate the development of multimodal interventions combining exercise training with pharmacological therapies, nutritional strategies, and behavioral interventions. Such an integrated approach has the potential not only to improve functional recovery but also to modify the long-term cardiovascular trajectory of individuals recovering from SARS-CoV-2 infection. As the population of COVID-19 survivors continues to grow worldwide, generating high-quality evidence capable of informing evidence-based rehabilitation and cardiovascular prevention strategies should remain a major international research priority.

## Conclusion

Persistent cardiovascular abnormalities following COVID-19 are consistently associated with endothelial dysfunction, chronic low-grade inflammation, arterial stiffness, autonomic dysregulation, metabolic impairment, and physical deconditioning. The evidence reviewed indicates that these interconnected mechanisms may contribute to vascular dysfunction and a pro-atherogenic phenotype after SARS-CoV-2 infection. Exercise-based rehabilitation has been shown to improve functional capacity, exercise tolerance, fatigue, dyspnea, quality of life, and several physiological parameters related to vascular and cardiometabolic health, including endothelial function, inflammatory status, autonomic regulation, insulin sensitivity, and arterial stiffness.

However, the available evidence does not yet demonstrate that exercise reverses accelerated vascular aging or reduces major adverse cardiovascular events in individuals with post-COVID syndrome. Furthermore, uncertainties remain regarding the optimal exercise prescription, the durability of vascular adaptations, and which patient subgroups derive the greatest benefit. Additional well-designed longitudinal and randomized studies incorporating objective vascular biomarkers and clinical cardiovascular outcomes are needed to clarify the long-term effects of exercise on cardiovascular risk after COVID-19

## Key References

- Bruno RM, Badhwar S, Abid L, et al. Accelerated vascular ageing after COVID-19 infection: the CARTESIAN study. Eur Heart J. 2025;46:3905–3918. doi: 10.1093/eurheartj/ehaf430.
  - This multicenter study provides strong evidence that COVID-19 is associated with persistent vascular aging and increased arterial stiffness, supporting the concept of accelerated cardiovascular aging after infection.
- Chidambaram V, Kumar A, Sadaf MI, et al. COVID-19 in the Initiation and Progression of Atherosclerosis. JACC Advances. 2024;3:101107. doi: 10.1016/j.jacadv.2024.101107.
  - This review summarizes the molecular and clinical evidence linking SARS-CoV-2 infection to endothelial dysfunction, vascular inflammation, and atherosclerotic progression.
- Du S, Cui Z, Xu X, et al. Clinical efficacy of exercise in the treatment of post-COVID-19 syndrome: a systematic review and network meta-analysis. Front Physiol. 2025;16:1656713. doi: 10.3389/fphys.2025.1656713.
  - This comprehensive network meta-analysis demonstrated that exercise interventions improve functional capacity, dyspnea, and quality of life in individuals with post-COVID syndrome.

## Data Availability

No datasets were generated or analysed during the current study.

## Abbreviations

ACE2: Angiotensin–converting enzyme 2
AIx: Augmentation Index
ANS: Autonomic Nervous System
CCL2: C–C motif chemokine ligand 2
CPET: Cardiopulmonary Exercise Testing
CRP: C–reactive Protein
CXCL8: C–X–C motif chemokine ligand 8
eNOS: Endothelial Nitric Oxide Synthase
FMD: Flow–Mediated Dilation
GLUT-4: Glucose Transporter Type 4
HDL: High–Density Lipoprotein
HIF-2α: Hypoxia–Inducible Factor–2 Alpha
HRV: Heart Rate Variability
IFN-γ: Interferon Gamma
IL-1RA: Interleukin–1 Receptor Antagonist
IL-6: Interleukin–6
IL-8: Interleukin–8
IL-10: Interleukin–10
LDL: Low–Density Lipoprotein
MAPK: Mitogen–Activated Protein Kinase
MCP-1: Monocyte Chemoattractant Protein–1
MeSH: Medical Subject Headings
NADPH: Nicotinamide Adenine Dinucleotide Phosphate
NLR: Neutrophil–to–Lymphocyte Ratio
NO: Nitric Oxide
PASC: Post–Acute Sequelae of SARS–CoV–2 Infection
POTS: Postural Orthostatic Tachycardia Syndrome
PWV: Pulse Wave Velocity
RCT: Randomized Controlled Trial
ROS: Reactive Oxygen Species
SARS-CoV–2: Severe Acute Respiratory Syndrome Coronavirus 2
TLR4: Toll–Like Receptor 4
TNF-α: Tumor Necrosis Factor Alpha
VEGF: Vascular Endothelial Growth Factor
VO₂peak: Peak Oxygen Uptake

## References

[1] Griffin DO. Postacute sequelae of COVID (PASC or Long COVID): an evidenced-based approach. Open Forum Infect Dis. 2024;11(9). 10.1093/ofid/ofae462. 10.1093/ofid/ofae462 PMC11363684 39220656

[2] Davis HE, McCorkell L, Vogel JM, Topol EJ. Long COVID: major findings, mechanisms and recommendations. Nat Rev Microbiol. 2023;21(3):133–46. 10.1038/s41579-022-00846-2. 10.1038/s41579-022-00846-2 PMC9839201 36639608

[3] Sinclair JE, Vedelago C, Ryan FJ, Carney M, Redd MA, Lynn MA, et al. Post-acute sequelae of SARS-CoV-2 cardiovascular symptoms are associated with trace-level cytokines that affect cardiomyocyte function. Nat Microbiol. 2024;9(12):3135–47. 10.1038/s41564-024-01838-z. 39478108 10.1038/s41564-024-01838-z PMC11602718

[4] Singh TK, Zidar DA, McCrae K, Highland KB, Englund K, Cameron SJ, et al. A post-pandemic enigma: the cardiovascular impact of post-acute sequelae of SARS-CoV-2. Circ Res. 2023;132(10):1358–73. 10.1161/CIRCRESAHA.122.322228. 37167358 10.1161/CIRCRESAHA.122.322228 PMC10171306

[5] Ribeiro GJS, Pinto A de A, Souza GC, Moriguchi EH. Association between pre-existing cardiovascular risk factors and post-acute sequelae of COVID-19 in older adults. An Sist Sanit Navar. 2025;48(1). 10.23938/ASSN.1103, 10.23938/ASSN.1103 PMC11925477 39949251

[6] Bruno RM, Badhwar S, Abid L, Agharazii M, Anastasio F, Bellien J, et al. Accelerated vascular ageing after COVID-19 infection: the CARTESIAN study. Eur Heart J. 2025;46(39):3905–18. 10.1093/eurheartj/ehaf430. 40819656 10.1093/eurheartj/ehaf430 PMC12517754

[7] Stojanovic M, Djuric M, Nenadic I, Bojic S, Andrijevic A, Popovic A, et al. Vascular Complications of Long COVID—From Endothelial Dysfunction to Systemic Thrombosis: A Systematic Review. Int J Mol Sci. 2025;27(1):433. 10.3390/ijms27010433. 10.3390/ijms27010433 PMC12786942 41516301

[8] Zhang X, Liu J, Deng X, Bo L. Understanding COVID-19-associated endothelial dysfunction: role of PIEZO1 as a potential therapeutic target. Front Immunol. 2024;15. 10.3389/fimmu.2024.1281263. 10.3389/fimmu.2024.1281263 PMC10937424 38487535

[9] Aljadah M, Khan N, Beyer AM, Chen Y, Blanker A, Widlansky ME. Clinical implications of COVID-19-Related endothelial dysfunction. JACC: Advances. 2024;3(8):101070. 10.1016/j.jacadv.2024.101070. 10.1016/j.jacadv.2024.101070 PMC11269277 39055276

[10] Yanai H, Adachi H, Hakoshima M, Katsuyama H, Sako A. The significance of endothelial dysfunction in long COVID-19 for the possible future pandemic of chronic kidney disease and cardiovascular disease. Biomolecules. 2024;14(8):965. 10.3390/biom14080965. 10.3390/biom14080965 PMC11352301 39199353

[11] Santos-de-Araújo AD, de Oliveira Garcia BR, Bassi-Dibai D, Júnior NFS, Ricci PA, Camargo PF, et al. A single bout of submaximal aerobic functional capacity test acutely promotes endothelial function in long COVID patients. Sci Rep. 2026;16(1):10439. 10.1038/s41598-026-41182-2. 41741603 10.1038/s41598-026-41182-2 PMC13031956

[12] Ferrari F, Goulart C, Franzoni LT, Cipriano G, Stein R. Effects of different exercise training modalities in post-COVID-19 individuals: a systematic review of randomized controlled trials. Disabil Rehabil. 2026. 10.1080/09638288.2026.2619815. 41622853 10.1080/09638288.2026.2619815

[13] Yanhui L, Jiangang W, Jianping G, Tao W. Post-COVID-19 syndrome and low-grade inflammation: exploring gender and occupational inequalities in a retrospective cohort study. Int Health. 2025;17(5):720–6. 10.1093/inthealth/ihaf021. 10.1093/inthealth/ihaf021 PMC12406786 40138630

[14] Gentilotti E, Alvarez Garavito C, Górska A, Gusinow R, Canziani LM, De Nardo P, et al. Persistent low-grade inflammation and post-COVID condition: evidence from the ORCHESTRA cohort. Biomedicines. 2025;14(1):83. 10.3390/biomedicines14010083. 10.3390/biomedicines14010083 PMC12839268 41595620

[15] Maamar M, Artime A, Pariente E, Fierro P, Ruiz Y, Gutiérrez S, et al. Post-COVID-19 syndrome, low-grade inflammation and inflammatory markers: a cross-sectional study. Curr Med Res Opin. 2022;38(6):901–9. 10.1080/03007995.2022.2042991. 35166141 10.1080/03007995.2022.2042991 PMC8935459

[16] Suárez-Reyes A, Villegas-Valverde CA. Implications of Low-grade Inflammation in SARS-CoV-2 Immunopathology. MEDICC Rev. 2021;23(2). 10.37757/MR2021.V23.N2.4. 10.37757/MR2021.V23.N2.4 33974614

[17] Elahi R, Karami P, Heidary AH, Esmaeilzadeh A. An updated overview of recent advances, challenges, and clinical considerations of IL-6 signaling blockade in severe coronavirus disease 2019 (COVID-19). Int Immunopharmacol. 2022;105:108536. 10.1016/j.intimp.2022.108536. 35074571 10.1016/j.intimp.2022.108536 PMC8747952

[18] Chidambaram V, Kumar A, Sadaf MI, Lu E, Al’Aref SJ, Tarun T, et al. COVID-19 in the initiation and progression of atherosclerosis. JACC: Advances. 2024;3(8):101107. 10.1016/j.jacadv.2024.101107. 10.1016/j.jacadv.2024.101107 PMC11304887 39113913

[19] Alsaidan AA, Al-Kuraishy HM, Al‐Gareeb AI, Alexiou A, Papadakis M, Alsayed KA, et al. The potential role of SARS‐CoV‐2 infection in acute coronary syndrome and type 2 myocardial infarction (T2MI): Intertwining spread. Immun Inflamm Dis. 2023;11(3). 10.1002/iid3.798. 10.1002/iid3.798 PMC10022425 36988260

[20] Bonaventura A, Vecchié A, Dagna L, Martinod K, Dixon DL, Van Tassell BW, et al. Endothelial dysfunction and immunothrombosis as key pathogenic mechanisms in COVID-19. Nat Rev Immunol. 2021;21(5):319–29. 10.1038/s41577-021-00536-9. 10.1038/s41577-021-00536-9 PMC8023349 33824483

[21] Xu S wen, Ilyas I, Weng J ping. Endothelial dysfunction in COVID-19: an overview of evidence, biomarkers, mechanisms and potential therapies. Acta Pharmacol Sin. 2023;44(4):695–709. 10.1038/s41401-022-00998-0. 10.1038/s41401-022-00998-0 PMC9574180 36253560

[22] Perico L, Benigni A, Casiraghi F, Ng LFP, Renia L, Remuzzi G. Immunity, endothelial injury and complement-induced coagulopathy in COVID-19. Nat Rev Nephrol. 2021;17(1):46–64. doi:10.1038/s41581-020-00357-4.. 10.1038/s41581-020-00357-4 PMC7570423 33077917

[23] Muir KC, Harris DD, Kanuparthy M, Hu J, Nho JW, Stone C, et al. Cellular and molecular mechanisms of SARS-CoV-2 spike protein-induced endothelial dysfunction. Cells. 2026;15(3):234. 10.3390/cells15030234. 10.3390/cells15030234 PMC12896700 41677601

[24] Ribeiro A, Wallraven T, Lech M, Adorjan K, Stubbe HC, Seifert M, et al. SARS−CoV−2 spike S1-mediated HIF−2α activation in retinal endothelial cells suggests a mechanism contributing to post−COVID endothelial dysfunction. Front Immunol. 2026. 10.3389/fimmu.2026.1770758. 41878417 10.3389/fimmu.2026.1770758 PMC13006635

[25] Norooznezhad AH, Mansouri K. Endothelial cell dysfunction, coagulation, and angiogenesis in coronavirus disease 2019 (COVID-19). Microvasc Res. 2021;137:104188. 10.1016/j.mvr.2021.104188. 34022205 10.1016/j.mvr.2021.104188 PMC8135191

[26] Balan OV, Malysheva IE, Tikhonovich EL, Lysenko LA. Dysregulation of MMP-2 and MMP-9 in post-COVID-19 and IPF: correlations with systemic inflammation and endothelial dysfunction. J Clin Med. 2026;15(2):671. 10.3390/jcm15020671. 41598609 10.3390/jcm15020671 PMC12842469

[27] Dimosiari A, Patoulias D. Effect of COVID-19 on endothelial function evaluated with flow-mediated dilation: another prognostic marker? A meta-analysis of observational studies. Arch Med Sci Ateroscler Dis. 2022;7(1):63–5. 10.5114/amsad/150638. 10.5114/amsad/150638 PMC9487834 36158065

[28] Aljadah M, Khan N, Beyer AM, Chen Y, Blanker A, Widlansky ME. Clinical implications of COVID-19-related endothelial dysfunction. JACC: Advances. 2024;3(8):101070. 10.1016/j.jacadv.2024.101070. 10.1016/j.jacadv.2024.101070 PMC11269277 39055276

[29] Stojanovic M, Djuric M, Nenadic I, Bojic S, Andrijevic A, Popovic A, et al. Vascular complications of long COVID—From endothelial dysfunction to systemic thrombosis: a systematic review. Int J Mol Sci. 2025;27(1):433. 10.3390/ijms27010433. 10.3390/ijms27010433 PMC12786942 41516301

[30] Jones AE, Khan Z, McGroder CF, Murphy SO, Depender C, Mira-Sanchez M, et al. Post-acute sequelae of COVID-19 persist over 3 years in acute lung injury/acute respiratory distress syndrome survivors but are not associated with persistent thromboinflammation or endothelial dysfunction. Crit Care Explor. 2026;8(3):e1390. 10.1097/CCE.0000000000001390. 10.1097/CCE.0000000000001390 PMC12987405 41824803

[31] Jannasz I, Pruc M, Rahnama-Hezavah M, Targowski T, Olszewski R, Feduniw S, et al. The impact of COVID-19 on carotid–femoral pulse wave velocity: a systematic review and meta-analysis. J Clin Med. 2023;12(17):5747. 10.3390/jcm12175747. 10.3390/jcm12175747 PMC10488425 37685813

[32] Schnaubelt S, Oppenauer J, Tihanyi D, Mueller M, Maldonado-Gonzalez E, Zejnilovic S, et al. Arterial stiffness in acute COVID‐19 and potential associations with clinical outcome. J Intern Med. 2021;290(2):437–43. 10.1111/joim.13275. 10.1111/joim.13275 PMC8013324 33651387

[33] Stamatelopoulos K, Georgiopoulos G, Baker KF, Tiseo G, Delialis D, Lazaridis C, et al. Estimated pulse wave velocity improves risk stratification for all-cause mortality in patients with COVID-19. Sci Rep. 2021;11(1):20239. 10.1038/s41598-021-99050-0. 10.1038/s41598-021-99050-0 PMC8511157 34642385

[34] Bruno RM, Badhwar S, Abid L, Agharazii M, Anastasio F, Bellien J, et al. Accelerated vascular ageing after COVID-19 infection: the CARTESIAN study. Eur Heart J. 2025;46(39):3905–18. 10.1093/eurheartj/ehaf430. 10.1093/eurheartj/ehaf430 PMC12517754 40819656

[35] Jakobs K, Reinshagen L, Puccini M, Friebel J, Wilde ACB, Alsheik A, et al. Disease severity in moderate-to-severe COVID-19 Is Associated with platelet hyperreactivity and innate immune activation. Front Immunol. 2022;13. 10.3389/fimmu.2022.844701. 10.3389/fimmu.2022.844701 PMC8963244 35359931

[36] Martins-Gonçalves R, Campos MM, Palhinha L, Azevedo-Quintanilha IG, Abud Mendes M, Ramos Temerozo J, et al. Persisting platelet activation and hyperactivity in COVID-19 survivors. Circ Res. 2022;131(11):944–7. 10.1161/CIRCRESAHA.122.321659. 10.1161/CIRCRESAHA.122.321659 PMC9645447 36268747

[37] Balmakov Y, Mark T, Barnett I, Cipok M, Lev EI, Cohen A, et al. Immature platelets and platelet reactivity in patients with COVID-19. clinical and applied thrombosis/hemostasis. 2025;31. 10.1177/10760296251318320. 10.1177/10760296251318320 PMC11826839 39943824

[38] Man DE, Andor M, Buda V, Kundnani NR, Duda-Seiman DM, Craciun LM, et al. Insulin Resistance in Long COVID-19 Syndrome. J Pers Med. 2024;14(9):911. 10.3390/jpm14090911. 10.3390/jpm14090911 PMC11433386 39338165

[39] 39. Chen M, Zhu B, Chen D, Hu X, Xu X, Shen WJ, et al. COVID-19 May increase the risk of insulin resistance in adult patients without diabetes: a 6-month prospective study. Endocrine Practice. 2021;27(8):834–41. doi:10.1016/j.eprac.2021.04.004. 10.1016/j.eprac.2021.04.004 PMC8054613 33887468

[40] Fierro P, Martín D, Pariente-rodrigo E, Pini SF, Basterrechea H, Tobalina M, et al. Post-COVID-19 syndrome, inflammation and insulin resistance: a retrospective cohort study. Minerva Endocrinology. 2025;50(2). 10.23736/S2724-6507.23.04108-8. 10.23736/S2724-6507.23.04108-8 38656093

[41] Xu Y, Xu JW, Wu Y, Rong LJ, Ye L, Franco OH, et al. Prevalence and prognosis of sarcopenia in acute COVID-19 and long COVID: a systematic review and meta-analysis. Ann Med. 2025;57(1). 10.1080/07853890.2025.2519678. 10.1080/07853890.2025.2519678 PMC12931308 40552782

[42] Piotrowicz K, Gąsowski J, Michel JP, Veronese N. Post-COVID-19 acute sarcopenia: physiopathology and management. Aging Clin Exp Res. 2021;33(10):2887–98. 10.1007/s40520-021-01942-8. 10.1007/s40520-021-01942-8 PMC8323089 34328636

[43] Földi M, Farkas N, Kiss S, Dembrovszky F, Szakács Z, Balaskó M, et al. Visceral adiposity elevates the risk of critical condition in COVID-19: a systematic review and meta‐analysis. Obesity. 2021;29(3):521–8. 10.1002/oby.23096. 10.1002/oby.23096 PMC7753720 33263191

[44] Scheffler M, Genton L, Graf CE, Remuinan J, Gold G, Zekry D, et al. Prognostic role of subcutaneous and visceral adiposity in hospitalized octogenarians with COVID-19. J Clin Med. 2021;10(23):5500. 10.3390/jcm10235500. 10.3390/jcm10235500 PMC8658645 34884199

[45] Belli S, Balbi B, Prince I, Cattaneo D, Masocco F, Zaccaria S, et al. Low physical functioning and impaired performance of activities of daily life in COVID-19 patients who survived hospitalisation. European Respiratory Journal. 2020;56(4):2002096. 10.1183/13993003.02096-2020. 10.1183/13993003.02096-2020 PMC7411272 32764112

[46] Akimoto K, Asano S, Satoh Y, Yokogushi K. Changes in respiratory, physical, and mental conditions in moderate and severe COVID-19 cases at our convalescent rehabilitation ward. Japanese Journal of Comprehensive Rehabilitation Science. 2023;14(0):10–5. 10.11336/jjcrs.14.10. 10.11336/jjcrs.14.10 PMC10585009 37859788

[47] Bakali M, Ward TC, Daynes E, Jones A V, Hawthorne GM, Latimer L, et al. Effect of aerobic exercise training on pulse wave velocity in adults with and without long-term conditions: a systematic review and meta-analysis. Open Heart. 2023;10(2):e002384. 10.1136/openhrt-2023-002384. 10.1136/openhrt-2023-002384 PMC10729135 38101857

[48] Xi H, Du L, Li G, Zhang S, Li X, Lv Y, et al. Effects of exercise on pulse wave velocity in hypertensive and prehypertensive patients: a systematic review and meta-analysis of randomized controlled trials. Front Cardiovasc Med. 2025;12. 10.3389/fcvm.2025.1504632. 10.3389/fcvm.2025.1504632 PMC11872916 40034990

[49] Du S, Cui Z, Xu X, Liu T, Ye J. Clinical efficacy of exercise in the treatment of post-COVID-19 syndrome: a systematic review and network meta-analysis. Front Physiol. 2025;16. 10.3389/fphys.2025.1656713. 10.3389/fphys.2025.1656713 PMC12756122 41488929

[50] Tartibian B, Khayat SMA, Maleki BH, Chehrazi M. The Effects of Exercise Training on Recovery of Biochemical and Hematological Outcomes in Patients Surviving COVID-19: A Randomized Controlled Assessor-Blinded Trial. Sports Med Open. 2022;8(1):152. 10.1186/s40798-022-00546-4. 10.1186/s40798-022-00546-4 PMC9782268 36562867

[51] Filgueira TO, Carvalho PRC, de Sousa Fernandes MS, Castoldi A, Teixeira AM, de Albuquerque RB, et al. The impact of supervised physical exercise on chemokines and cytokines in recovered COVID-19 patients. Front Immunol. 2023;13. 10.3389/fimmu.2022.1051059. 10.3389/fimmu.2022.1051059 PMC9846636 36685603

[52] de Castro GS, Gama LR, Ramos AF, Gatti da Silva G, Teixeira AA de S, Cunha-Neto E, et al. Post-COVID-19 condition: systemic inflammation and low functional exercise capacity. Front Nutr. 2024;11. 10.3389/fnut.2024.1295026. 10.3389/fnut.2024.1295026 PMC10973152 38549752

[53] Batatinha HAP, Krüger K, Rosa Neto JC. Thromboinflammation and COVID-19: the role of exercise in the prevention and treatment. Front Cardiovasc Med. 2020;7. 10.3389/fcvm.2020.582824. 10.3389/fcvm.2020.582824 PMC7775570 33392268

[54] Pelle MC, Zaffina I, Lucà S, Forte V, Trapanese V, Melina M, et al. Endothelial dysfunction in COVID-19: potential mechanisms and possible therapeutic options. 2022;12(10):1605. 10.3390/life12101605. 10.3390/life12101605 PMC9604693 36295042

[55] Montiel V, Lobysheva I, Gérard L, Vermeersch M, Perez-Morga D, Castelein T, et al. Oxidative stress-induced endothelial dysfunction and decreased vascular nitric oxide in COVID-19 patients. EBioMedicine. 2022;77:103893. 10.1016/j.ebiom.2022.103893. 10.1016/j.ebiom.2022.103893 PMC8865837 35219085

[56] Shivgulam ME, Liu H, Schwartz BD, Langley JE, Bray NW, Kimmerly DS, et al. Impact of exercise training interventions on flow-mediated dilation in adults: an umbrella review. Sports Medicine. 2023;53(6):1161–74. 10.1007/s40279-023-01837-w. 10.1007/s40279-023-01837-w 37017797

[57] Tao X, Chen Y, Zhen K, Ren S, Lv Y, Yu L. Effect of continuous aerobic exercise on endothelial function: A systematic review and meta-analysis of randomized controlled trials. Front Physiol. 2023;14. 10.3389/fphys.2023.1043108. 10.3389/fphys.2023.1043108 PMC9950521 36846339

[58] Ambrosino P, Parrella P, Formisano R, Perrotta G, D’Anna SE, Mosella M, et al. Cardiopulmonary exercise performance and endothelial function in convalescent COVID-19 patients. J Clin Med. 2022;11(5):1452. 10.3390/jcm11051452.. 10.3390/jcm11051452 PMC8911200 35268542

[59] Choi TG, Kim JY, Seong JY, Min HJ, Jung YJ, Kim YW, et al. Impaired endothelial function in individuals with post-acute sequelae of COVID-19. J Cardiopulm Rehabil Prev. 2025;45(2):146–52. 10.1097/HCR.0000000000000928. 10.1097/HCR.0000000000000928 40014640

[60] Bil J, Możeńska O. The vicious cycle: a history of obesity and COVID-19. BMC Cardiovasc Disord. 2021;21(1):332. 34229605 10.1186/s12872-021-02134-y PMC8258476

[61] Yong SJ. Long COVID or post-COVID-19 syndrome: putative pathophysiology, risk factors, and treatments. Infect Dis. 2021;53(10):737–54. 10.1080/23744235.2021.1924397 PMC8146298 34024217

[62] Ashton R, Ansdell P, Hume E, Maden-Wilkinson T, Ryan D, Tuttiett E, et al. COVID-19 and the long-term cardio-respiratory and metabolic health complications. Rev Cardiovasc Med. 2022;23(2):53. 35229544 10.31083/j.rcm2302053

[63] Mińko A, Turoń-Skrzypińska A, Rył A, Rotter I. The impact of metabolic syndrome on the outcomes of rehabilitation in post-COVID-19 patients. J Clin Med. 2025;14(16):5893. 40869721 10.3390/jcm14165893 PMC12387359

[64] Garbsch R, Schäfer H, Mooren FC, Schmitz B. Analysis of fat oxidation capacity during cardiopulmonary exercise testing indicates long-lasting metabolic disturbance in patients with post-COVID-19 syndrome. Clin Nutr. 2024;43(12):26–35. 39423759 10.1016/j.clnu.2024.10.010

[65] Wu ZJ, Han C, Wang ZY, Li FH. Combined training prescriptions for improving cardiorespiratory fitness, physical fitness, body composition, and cardiometabolic risk factors in older adults: systematic review and meta-analysis of controlled trials. Sci Sports. 2024;39(1):1–18. 10.1016/j.scispo.2022.03.015. 10.1016/j.scispo.2022.03.015 PMC9937425 36843900

[66] Al-Mhanna SB, Batrakoulis A, Mohamed M, Alkhamees NH, Sheeha B Bin, Ibrahim ZM, et al. Home-based circuit training improves blood lipid profile, liver function, musculoskeletal fitness, and health-related quality of life in overweight/obese older adult patients with knee osteoarthritis and type 2 diabetes: a randomized controlled trial during the COVID-19 pandemic. BMC Sports Sci Med Rehabil. 2024;16(1):125. 10.1186/s13102-024-00915-4. 10.1186/s13102-024-00915-4 PMC11145895 38831437

[67] Abdulan IM, Feller V, Oancea A, Maștaleru A, Alexa AI, Negru R, et al. Evolution of cardiovascular risk factors in post-COVID patients. J Clin Med. 2023;12(20):6538. 37892676 10.3390/jcm12206538 PMC10607829

[68] Menezes D, Lima P, Lima I, Uesugi JHE, Vasconcelos P, Quaresma JAS, et al. Metabolic profile of patients with long COVID: a cross-sectional study. Nutrients. 2023;15(5):1197. 36904195 10.3390/nu15051197 PMC10005061

[69] Di Girolamo FG, Fiotti N, Sisto UG, Nunnari A, Colla S, Mearelli F, et al. Skeletal muscle in hypoxia and inflammation: insights on the COVID-19 pandemic. Front Nutr. 2022;9:865402. 10.3389/fnut.2022.865402 PMC9072827 35529457

[70] Rezel-Potts E, Douiri A, Sun X, Chowienczyk PJ, Shah AM, Gulliford MC. Cardiometabolic outcomes up to 12 months after COVID-19 infection. A matched cohort study in the UK. PLoS Med. 2022;19(7):e1004052. 35853019 10.1371/journal.pmed.1004052 PMC9295991

